# Correlation between schistosomiasis and CD8+ T cell and stromal PD-L1 as well as the different prognostic role of CD8+ T cell and PD-L1 in schistosomal-associated colorectal cancer and non-schistosomal-associated colorectal cancer

**DOI:** 10.1186/s12957-021-02433-w

**Published:** 2021-11-07

**Authors:** Weixia Wang, Hongyan Jing, Jican Liu, Dacheng Bu, Yingyi Zhang, Ting Zhu, Kui Lu, Yanchao Xu, Meihong Cheng, Jing Liu, Junxia Yao, Sinian Huang, Limei Wang

**Affiliations:** Department of Pathology, Qingpu Branch of Zhongshan Hospital, Fudan University, No. 1158 East Park Road, Qingpu District, Shanghai, 201700 People’s Republic of China

**Keywords:** PD-L1, CD8+ TILs, Colorectal cancer, Prognosis, Schistosomiasis

## Abstract

**Background:**

The effect of schistosomiasis on CD8+ T cells and then on PD-L1 expression was unknown, and the utility of CD8+ TILs as a biomarker for schistosomal-associated colorectal cancer (SCRC) rarely has been reported.

**Methods:**

Three hundred thirty-eight patients with colorectal cancer (CRC) were enrolled. Immunohistochemical analysis was conducted to evaluate the expression of PD-L1 and the infiltration of CD8+ T cells.

**Results:**

In the total cohort, the results showed that CD8^+^ TIL density was positively correlated with tumoral (*p* = 0.0001) and stromal PD-L1 expression (*p* = 0.0102). But there were no correlation between schistosomiasis and CD8+ TILs and PD-L1. Furthermore, CD8^+^ TIL density (*p* = 0.010), schistosomiasis (*p* = 0.042) were independent predictive factors for overall survival (OS). Stromal PD-L1 (sPD-L1) was correlated with OS (*p = 0.046*), but it was not an independent predictor. In patients without schistosomiasis, CD8 + T cells (*p* = 0.002) and sPD-L1 (*p* = 0.005) were associated with better OS. In patients with schistosomiasis, CD8 + T cells were independent prognosis factor (*p* = 0.045).

**Conclusions:**

The study showed that CD8+ TILs was an independent predictive factor for OS in CRC and SCRC patients. The expression of PD-L1 was positively associated with CD8 + TILs density. There were no correlation between schistosomiasis and CD8 + TILs and PD-L1. Stromal PD-L1 but not tPD-L1 was significantly associated with OS, whereas it was not an independent prognostic factor.

**Supplementary Information:**

The online version contains supplementary material available at 10.1186/s12957-021-02433-w.

## Introduction

Colorectal cancer is one of the most common malignant diseases worldwide. Although a variety of anticancer drugs have been developed, the death rates of CRC have not been obviously decreased [1, 2]. Expression of PD-L1 in intratumoral compartment has been suggested to influence immune response [3] and serve as a prognostic marker in CRC [4]. PD-L1 is not solely considered as a result of an increased immune inhibiting PD/PD-L1 interplay but rather is viewed as a reflection of adaptive antitumor immunity, where tumor-infiltrating lymphocytes are activated in response to tumor antigens [4]. It has been reported that PD-L1 on either tumor cells or host immune cells contributes to tumor escape, and the relative contributions of PD-L1 on these cells seem to be context-dependent [5]. Recent study showed that tumoral PD-L1 is a favorable prognostic factor in early stage of non-small cell carcinoma [6]. It was also reported that there were differences in outcome in triple-negative breast cancer depending on the expression of PD-L1 in the tumoral cell membrane, cytoplasm, and stromal cellular compartments [7]. Yaqi Li et al. reported that tumoral PD-L1 correlated with better prognosis of CRC patients [8]. Whereas some studies found that PD-L1 was associated with deleterious effect on survival [9, 10], these studied did not distinguish PD-L1 expression in tumoral or stromal cells. Therefore, PD-L1 expression used as a predictor factor is also controversial.

Studies reported that CD8+ TIL induces PD-L1 expression in tumor cells by producing IFNγ [11–13]. CD8+ T cells are thought to have antitumor functions during tumor development in a tumor microenvironment. Evidence has shown that activated CD8^+^ cytotoxic T lymphocytes were correlated with favorable survival of CRC patients and gastric cancer patients [14–17]. Therefore, further detailed analysis is needed to confirm the prognostic significance of PD-L1 and CD8+ TILs in CRC and to investigate the relationship between PD-L1 and CD8+ T cells.

The Qingpu District of Shanghai in China was one of the endemic areas. Schistosomiasis, which is an infectious disease [18], is considered as a risk factor for CRC [19]. Schistosomiasis is correlated with inflammation [20–22]. CD8+ TILs are the main force involved in inflammatory response. In addition, PD-L1 was involved in immune microenvironment and upregulated by CD8+ TILs. With these considerations, we wonder to investigate the relationship between schistosomiasis and CD8+ TILs and PD-L1.

In short, this study aimed primarily to investigate the effect of schistosoma infection on CD8+ TILs and PD-L1 expression and the relationship between schistosomiasis and CD8+ TILs and PD-L1 expression. Besides, we proposed to further to compare the prognostic role of PD-L1 and CD8+ TILs in SCRC and NSCRC.

## Methods and materials

### Patients

This retrospective analysis includes 338 patients with resected primary CRC at Qingpu Branch of Zhongshan Hospital affiliated to Fudan University, from January 2008 to August 2016. All of the operations followed the principle described previously [23]. The inpatient medical records and pathological reports were reviewed from the pathological system and Qingpu District Center for Disease Control and Prevention, and the patients were followed up by telephone. OS is defined as the interval from the surgical operation date to the last follow-up or death caused by CRC. Inclusion criteria are as previously described [23]. Two expert pathologists reviewed HE-stained slides to determine the diagnosis and to restage the tumors according to the eighth edition of American Joint Committee on Cancer (AJCC). This study is approved by the medical ethics committee of Fudan University, in accordance with the Helsinki Declaration of 1975. Prior written informed consent was obtained from all patients.

### Tissue microarrays (TMA)

The TMA blocks were manufactured from the most representative areas of individual paraffin blocks, as previously described [24]. Briefly, reviewed HE-stained slides and marked the represented areas in tumor tissues, and the single core (2 mm wide and 6 mm long) for each case was precisely arrayed into a new recipient paraffin block. The cores containing more than 20% tumor cells were considered as valid cores.

### Immunohistochemical (IHC)

All the tissue slides were stained by the fully automated Bond-III system (Leica Microsystems, Newcastle-upon-Tyne, UK) according to the manufacturer’s instructions. The following primary antibodies were used: PD-L1 (MXR003; 1:750; MXB Biotechnologies, Fuzhou, China) and CD8 (clone NCL-L-CD8-4B11; 1: 100; DAKO, Minneapolis, MN, USA).

### Pathological assessment of PD-L1 expression and CD8+ T cell density

PD-L1 IHC was analyzed independently by two experienced pathologists, who were unaware of the clinical data. The results were evaluated according to the percentage of the stained cells. Scoring was assessed in both tumoral membranous and stromal immune cell membranous compartments. Tumors were classified as PD-L1 positive if there was ≥ 1% tumoral membranous PD-L1 expression (tPD-L1^+^) or ≥ 1% stromal PD-L1 expression (sPD-L1^+^).

**Fig. 1:**
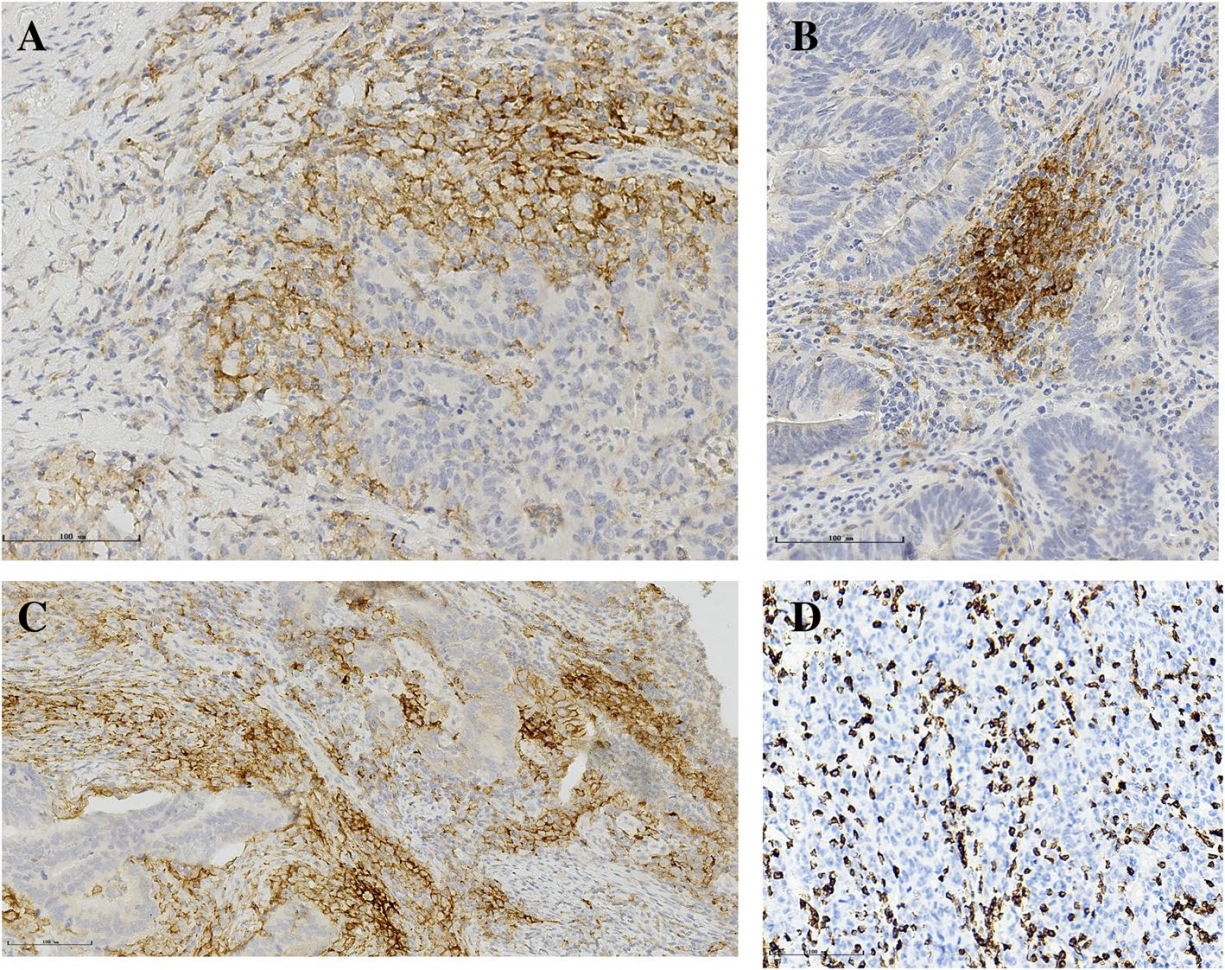
Immunohistochemical staining of representative programmed death-ligand 1 (PD-L1) expression (× 200) and CD8 (× 200) positivity. **A** PD-L1 expression positivity on tumor cells. **B** PD-L1 expression positivity on tumor-infiltrating mononuclear cells. **C** PD-L1 expression positivity both on tumor cells and within the immune stroma. **D** Immunohistochemical staining of representative CD8 positivity (× 200)

The TMA slides were scanned using a scanner system (PRECICE 500B) at × 40 magnification. For CD8, the densities of positively stained cells were evaluated on whole section slides using an image analysis system (Image J software, USA) (cells per square millimeter) (Fig. 1C). At least half of the core area was selected randomly, and the results of the calculated densities were extracted and put into an Excel file. Measurements were recorded as the mean number of positive cells per tissue unit in square millimeters as well as the number of positive cells among each 1-mm^2^ tissue units.

### Statistical analysis

Data were analyzed using SPSS (version 20.0; IBM Corp.) and Graphpad 5.0. Every variable was analyzed using univariate analysis to identify all potentially important predictors and then variables with *p* ≤ 0.05 in the univariate analysis were included in a multivariate analysis. Finally, multivariate Cox regression analysis was performed to identify predictive factors for OS.

## Results

### Patient characteristics

The clinical characteristics of the 338 patients are shown in Table 1. The median age of the patients at diagnosis was 67 years (range, 33–91 years). According to AJCC Staging Manual (seventh edition), there were very few highly differentiated cases in the follow-up data. Seventy-six percent cases were well/moderate differentiated, and 24% were poorly differentiated. Intriguingly, schistosoma infection was observed in 38% (128 out of 338) CRC patients (Supplementary Fig. 1). And the diagnosis of schistosomiasis was done by finding schistosome eggs in HE-stained slides.

**Table 1:** Clinicopathological characteristics of the CRC cohort

Characteristics	All patients (*N* = 338)	
	*N*	*%*
CD8^low^	104	*69*
tPD-L1^pos^	138	*41*
sPD-L1 ^pos^	200	*64*
Both tPD-L1^pos^ and sPD-L1 ^pos^	129	*38*
Age (< 60years)	83	*24*
Gender (male)	214	*61*
Tumor location		
Rectum	91	*27*
Left colon	112	*33*
Right colon	135	*40*
Tumor diameter (< 5 cm)	166	*49*
Tumor differentiation		
Well/moderate diff.	256	*76*
Poor diff.	82	*24*
Vessel invasion (present)	120	*36*
Intraneural invasion (present)	31	*0.9*
Tumor deposit (> 2 nodes)	42	*1.2*
Bowel perforation (present)	13	*0.4*
Tumor budding (≥ 5 buds)	215	*64*
Ulceration (yes)	145	*43*
Histological type		
Adenocarcinoma	297	*88*
Mucinous/SRCC	41	*12*
Pathological T stage		
T1-2	80	24
T3-4	258	76
Lymph node metastasis (yes)	140	*41*
TNM stage		
I + II	184	*54*
III+ IV	154	*46*
*Schistosomiasis* (positive)	128	*38*

CD8low = density ≤ 279 cell /mm2

*Abbreviations*: *CRC* colorectal cancer, *N* number, *SRCC* signet ring cell carcinoma

### Staining results of each marker

Figure 1 shows representative PD-L1-stained images on both tumor cells and tumor-infiltrating mononuclear cells. Among 338 cases analyzed, 41% of cases showed tumoral PD-L1 expression (tPD-L1^+^: defined as ≥ 1%), and 64% showed PD-L1 expression within the immune stroma (sPD-L1^+^: defined as ≥ 1%) (Table 1 and Fig. 1A, B). There were 38% (129 out of 338) of cases expressing PD-L1 both in tumoral and immune stroma (Table 1 and Fig. 1C). The median value of CD8^+^ density was 405 cell/mm^2^ (range, 0–2466 cell/mm^2^) (Table 1 and Fig. 1D).

### Relationship between schistosomiasis and CD8^+^ TIL density and PD-L1 expression

**Fig. 2:**
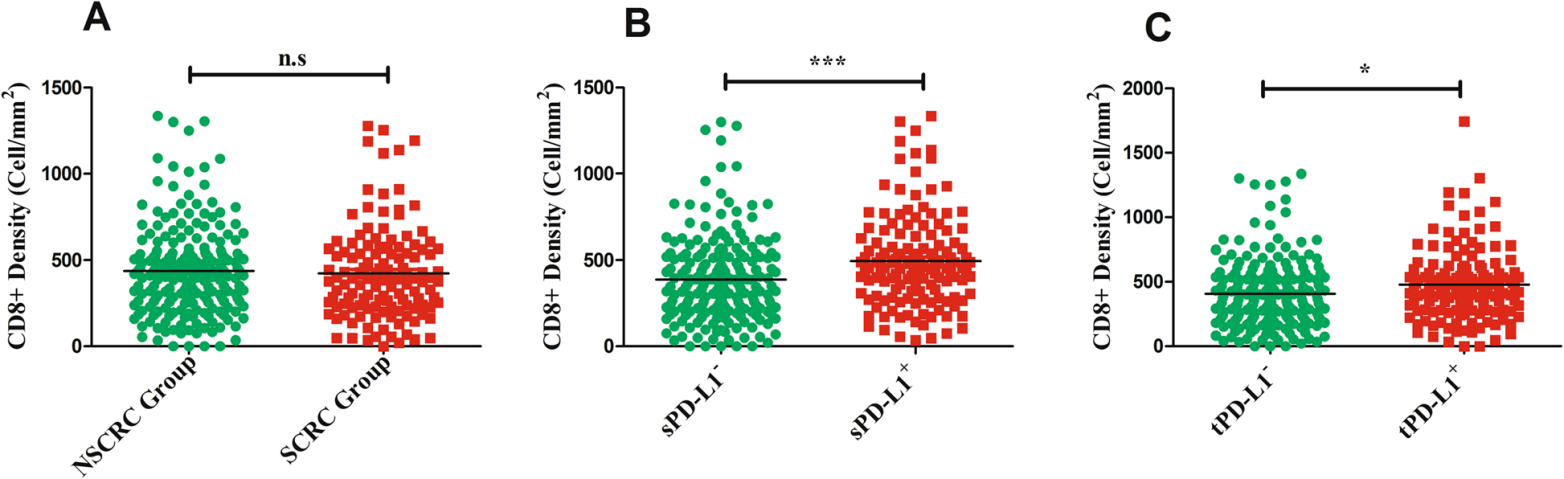
**A** The relationship between schistosomal infection and CD8+ TILs density (*p* > 0.05). **B** PD-L1 expression by immune stroma (*p* = 0.0001) increased with increasing CD8 density. **C** PD-L1 expression by tumor cells (*p* = 0.0102) increased with increasing CD8 density. Correlation between CD8 density and PD-L1 expression by location were examine using the Mann-Whitney test

Patients were divided into two groups: schistosomal-associated colorectal cancer (SCRC) patients and non-schistosomal-associated colorectal cancer (NSCRC) patients. As shown in Fig. 2A, there were no significant correlation between CD8+ TILs density and schistosomiasis (*p* > 0.05).

We next compared the correlation of CD8^+^ T cell density with PD-L1 on the tumor cells or in the immune stroma, respectively. As shown in Fig. 2B, C and Table 2, CD8^+^ T cell density was significantly higher within sPD-L1^+^ group than that within sPD-L1^-^ group (Fig. 2B, *p* < 0.0001) (sPD-L1^-^ group versus sPD-L1^+^ group, median 347 versus 460 cell/mm^2^), and it was also obviously higher within the tPD-L1^+^ group than within the tPD-L1^-^ group (Fig. 2C, *p* = 0.0102) (tPD-L1^+^ group versus tPD-L1^-^ group, median 371 versus 454 cell/mm^2^).

**Table 2:** The association between clinicopathological characteristics and PD-L1

Variables	No.	sPD-L1 expression		*p*		tPD-L1 expression		*p*
		Negative (*N* = 196)	Positive (*N* = 142)		No.	Negative (*N* = 200)	Positive (*N* = 138)	
Age				0.898				0.698
< 60	80	47 (24%)	33 (23%)		80	49 (25%)	31 (22%)	
≥ 60	258	149 (76%)	109 (77%)		258	151 (75%)	107 (78%)	
Gender				0.599				0.429
Male	205	120 (61%)	85 (60%)		133	125 (62%)	80 (58%)	
Female	133	76 (39%)	57 (40%)		205	75 (38%)	58 (42%)	
Tumor site				0.518				0.216
Rectum	91	49 (25%)	42 (30%)		91	52 (26%)	39 (28%)	
Left colon	112	68 (35%)	44 (31%)		112	74 (37%)	38 (28%)	
Right colon	135	79 (40%)	56 (39%)		135	74 (37%)	61 (44%)	
Tumor diameter				0.123				0.077
< 5 cm	166	89 (45%)	77 (53%)		166	90 (45%)	76 (55%)	
≥ 5 cm	172	107 (55%)	65 (47%)		172	110 (55%)	62 (45%)	
Tumor differentiation				0.797				0.521
Moderate	256	147 (75%)	109 (77%)		256	154 (76%)	102 (75%)	
Poor	82	49 (25%)	33 (23%)		82	46 (24%)	36 (25%)	
Pathological T stage				< 0.001^*^				0.177
I–II	77	28 (14%)	49 (35%)		65	39 (20%)	26 (19%)	
III	261	168 (86%)	93 (65%)		231	161 (80%)	70 (81%)	
Lymph node metastasis				0.034^*^				0.370
No	198	105 (54%)	93 (65%)		198	113 (57%)	85 (62%)	
Yes	140	91 (46%)	49 (35%)		140	87 (43%)	53 (38%)	
Tumor budding				0.039^*^				0.563
Negative (< 5 buds)	123	62 (32%)	61 (43%)		123	67 (34%)	56 (41%)	
Positive (≥ 5 buds)	215	134 (68%)	81 (57%)		215	133 (66%)	82 (59%)	
Vessel invasion				0.250				0.425
No	218	121 (62%)	97 (68%)		272	126 (63%)	91 (66%)	
Yes	120	75 (38%)	45 (32%)		67	74 (37%)	47 (34%)	
Intraneural invasion				0.849				0.614
No	307	177 (90%)	130 (92%)		307	181 (91%)	126 (91%)	
Yes	31	19 (10%)	12 (8%)		31	19 (9%)	12 (9%)	
Tumor deposit				0.018^*^				0.866
No	297	165 (84%)	132 (93%)		297	174 (87%)	123 (89%)	
Yes	41	31 (16%)	10 (7%)		41	26 (13%)	15 (11%)	
Histological type				0.314				0.471
Adenocarcinoma	297	169 (86%)	128 (90%)		297	175 (88%)	122 (88%)	
Mucinous/SRCC	41	27 (14%)	14 (10%)		41	25 (12%)	16 (12%)	
Bowel perforation				< 0.001^*^				0.859
No	325	185 (94%)	140 (99%)		332	192 (96%)	133 (96%)	
Yes	13	11 (6%)	2 (9%)		13	8 (4%)	5 (4%)	
Ulceration				0.825				0.738
No	193	113 (58%)	80 (56%)		193	116 (58%)	77 (56%)	
Yes	145	83 (42%)	62 (44%)		145	84 (42%)	61 (44%)	
TNM stage				0.037^*^				0.020^*^
I	185	97 (49%)	88 (61%)		184	104 (52%)	80 (58%)	
II								
III	155	99 (51%)	56 (39%)		138	96 (48%)	42 (42%)	
IV								
*Schistosomiasis*				0.650				0.210
Negative	210	124 (63%)	86 (61%)		210	130 (65%)	80 (58%)	
Positive	128	72 (37%)	56 (39%)		128	70 (35%)	58 (42%)	
CD8^+^ T cell density				0.001^*^				0.023^*^
Low group	1044	74 (38%)	30 (21%)		104	71 (36%)	33 (24%)	
High group	234	122 (62%)	112 (79%)		234	129 (64%)	105 (76%)	

— data is not applicable

*Abbreviations*: *sTILs* stromal tumor-infiltrating lymphocytes, *NSCRC* non-schistosomal colorectal cancer, *SCRC* schistosomal colorectal cancer, *N* Number, *LN* lymph node

The association between PD-L1 expression and clinicopathological characteristics was evaluated by using the chi-square and Fisher’s exact tests

### Correlation between PD-L1 expression and patient characteristics

The relationships of tPD-L1 and sPD-L1 expression with clinicopathologic features are detailed in Table 2. One hundred thirty-eight patients (41%) and 142 (42%) were placed in the tPD-L1^high^ (expression level ≥ 2%) and sPD-L1^high^ group (expression level ≥ 2%) based on the optimum cutoff point, respectively. Stromal PD-L1 positivity were significantly associated with less aggressive tumor features, including early pathological T stage (*p* < 0.001), absence of lymph node metastasis (*p* = 0.031), absence of tumor deposit (*p* = 0.012), early TNM Stage (*p* = 0.034), less tumor budding (*p* = 0.039), and less bowel perforation (*p* < 0.001). Meanwhile, tumoral PD-L1 positivity were significantly associated with early TNM Stage (*p* = 0.020) (Table 2).

### Prognostic significance of PD-L1 expression and CD8^+^ T cells density

**Fig. 3:**
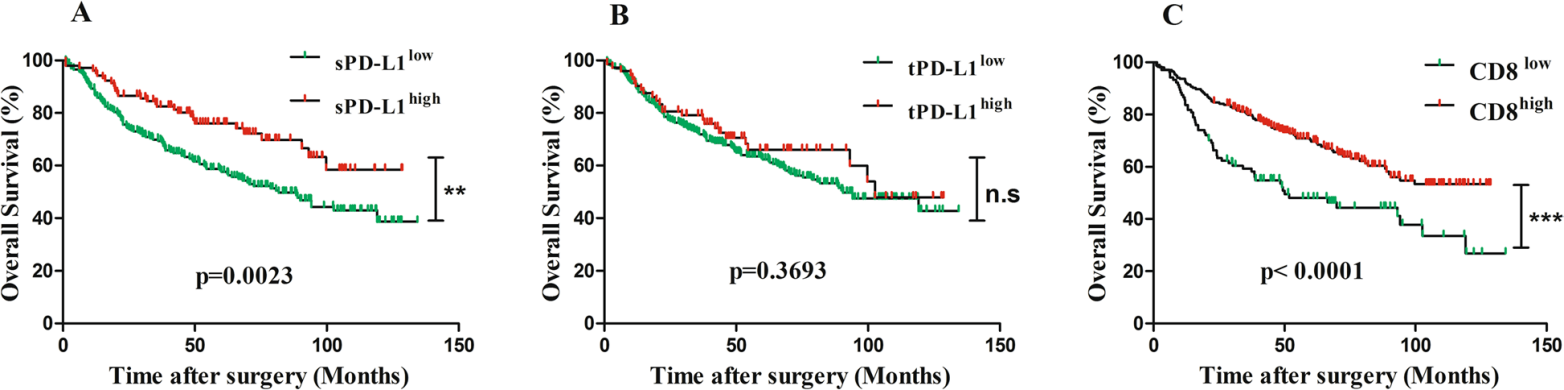
Kaplan-Meier curves for overall survival (OS) of CRC patients, OS was calculated using the Kaplan–Meier method and analyzed using the log-rank test. **A** OS of CRC patients with expression of PD-L1 on the immune stroma cells (sPD-L1 positive expressing ≥ 2%) (*p* = 0.0023). **B** OS of CRC patients with expression of PD-L1 on tumoral cells (tPD-L1 positive expressing ≥ 2%) (*p* = 0.3693). **C** OS of CRC patients with CD8+ cells density (*p < 0.0001*). The optimum cutoff value of CD8^+^T cell density were determined by X-tile program, which were 279 cell/mm^2^. CD8^low^ group was defined as CD8+ T cells density < 279, and CD8^high^ group was defined as CD8+ T cells density ≥ 279 cell/mm^2^

Mean and median time to OS was 62.54 and 62.85(1.25–134.4) months, respectively. During the follow-up, there were 42% (141 out of 338) patients died. Higher PD-L1 expression on both tumor cells (expression level ≥ 2%, tPD-L1^high^) and within the immune stroma (expression level ≥ 2%, sPD-L1^high^) was associated with better OS in CRC patients, but only the sPD-L1 reached statistical significance (*p* = 0.0023, Fig. 3A for sPD-L1; *p* = 0.3693, Fig. 3B for tPD-L1).

With regard to CD8^+^T cell, the optimum cutoff value, which was determined by X-tile program, was 279 cell/mm^2^ (Supplementary Fig. 2). Patients were divided into two groups for further analysis (CD8^low^ < 279 and CD8^high^ ≥ 279 cell/mm^2^). Tumors with higher CD8^+^ T cell density had better OS compared with that of with lower CD8^+^ T cell density (*p < 0.0001*, respectively, Fig. 3C).

The univariate Cox regression model indicated that age, gender, pathological T stage, lymph node metastasis, TNM stages, tumor differentiation, vessel invasion, tumor deposit, tumor budding, *Schistosomiasis*, CD8^+^ T cells, and sPD-L1 were significantly associated with OS (*p* < *0.05*, Table 3). Multivariate analysis after adjustment indicated that gender, TNM stage, tumor deposit, *Schistosomiasis*, and CD8^+^ T cells were independent prognostic factors for OS of CRC patients (*p* < 0.05, Table 3).

**Table 3:** Univariate and multivariate Cox regression of clinicopathological for overall survival

Variables	Univariate analysis		Multivariate analysis	
	*p*	HR (95% CI)	*p*	HR (95% CI)
Age (< 60years)	0.012	1.754 (1.129–2.726)		
Gender (male)	0.011	1.590 (1.112–2.272)	0.005	1.626 (1.133–2.335)
Tumor diameter (5 cm)	0.881	0.975 (0.669–1.360)		
Tumor site				
Rectum		Refer		
Left colon	0.906	1.026 (0.673–1.562)		
Right colon	0.438	0.849 (0.561–1.284)		
Pathological T stage	< 0.001	2.453 (1.477–4.074)		
Lymph node metastasis	< 0.001	2.891 (2.058–4.060)		
TNM stage	< 0.001	3.273 (2.305–4.649)	< 0.001	2.755 (1.887–4.022)
Tumor differentiation	0.002	1.775 (1.242–2.537)		
Vessel invasion	< 0.001	1.925 (1.376–2.692)		
Intraneural invasion	0.133	1.509 (0.882–2.584)		
Tumor deposit	< 0.001	4.095 (2.724–6.156)	< 0.001	2.102 (1.351–3.270)
Bowel perforation	0.815	0.888 (0.328–2.401)		
Tumor budding	< 0.001	1.856 (1.274–2.705)		
*Schistosomiasis*	0.048	1.388 (0.994–1.940)	0.042	1.424 (1.016–1.996)
Ulceration	0.554	0.903 (0.644–1.266)		
Histological type	0.521	1.168 (0.727–1.875)		
CD8 density	< 0.001	0.424 (0.294–0.611)	0.010	0.635 (0.449–0.897)
sPD-L1	0.046	0.702 (0.496–0.993)		
iPD-L1	0.540	0.637 (0.326–1.266)		

— data is non-significant

*Abbreviations*: *NSCRC* non-schistosomal colorectal cancer, *SCRC* schistosomal colorectal cancer, *CI* confidence interval, *HR* hazard ratio, *LN* lymph node

*p* < 0.05 was defined as the criterion for variable deletion when performing backward stepwise selection

### Survival analysis based on subgroups

**Fig. 4:**
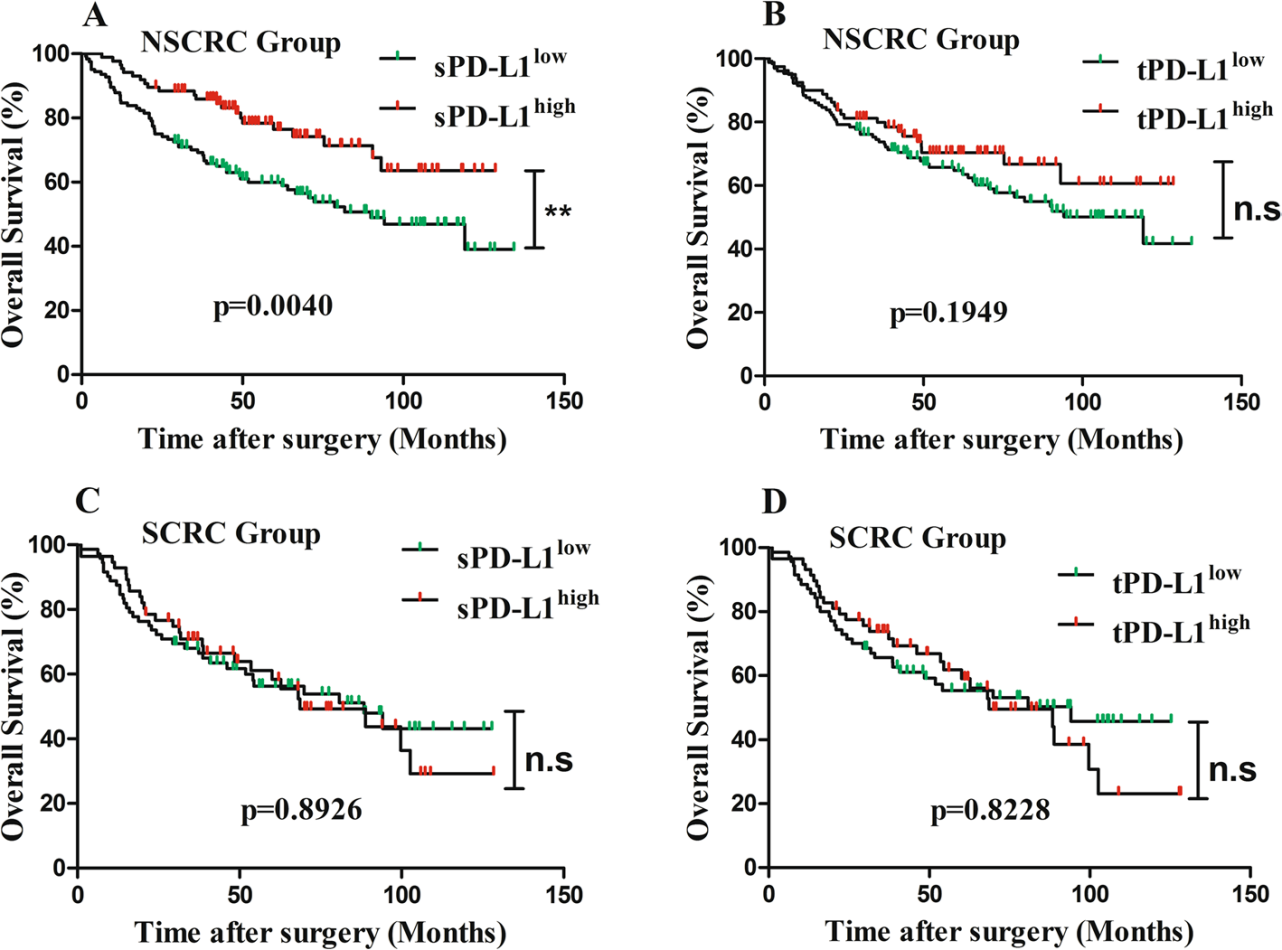
Kaplan-Meier curves for overall survival OS of CRC patients, OS was calculated using the Kaplan–Meier method and analyzed using the log-rank test. **A** PD-L1 expressing on the immune stroma cells (sPD-L1 positive expressing ≥ 2%) for OS of schistosomal-associated-colorectal cancer patients (SCRC) (*p* = 0.0040) or for OS of non-schistosomal-associated colorectal cancer patients (NSCRC) (**B**) (*p* = 0.1949). **C** PD-L1 expressing on the tumoral cells (tPD-L1 positive expressing ≥ 2%) for OS of SCRC patients (*p* = 0.8926) or for OS of NSCRC patients (**D**) (*p* = 0.8228)

Kaplan-Meier analysis demonstrated that merely sPD-L1 expression level was associated with favorable OS in the NSCRC group (*p = 0.0040*) (Fig. 4A), sPD-L1 expression level in the SCRC group and tPD-L1 in the both groups were not correlated with OS (*p* > *0.05*) (Fig. 4B–D). In the NSCRC set, the univariate Cox regression model revealed that gender, TNM stage, pathological T stage, lymph node metastasis, tumor differentiation, tumor budding, vessel invasion, tumor deposit, sPD-L1 expression level, and CD8^+^ T cells density were associated with OS (*p* < *0.05*) (Table 4), and the multivariate Cox regression analysis showed that gender, pathological T stage, TNM stage, tumor deposit, and CD8^+^ T cells density were independent prognosis factors (*p < 0.05*) (Table 4). In the SCRC set, the univariate analysis demonstrated that lymph node metastasis, TNM stage, tumor differentiation, tumor deposit, and CD8+ T cell density were associated with OS (*p* < *0.05*), and multivariate analysis results showed that only TNM stage, tumor deposit, and CD8+ T cell density were independent factors for OS (*p* < *0.05*).

**Table 4:** Univariate and multivariate analysis for overall survival in SCRC set and NSCRC set

Variables	SCRC set		NSCRC set	
	*p*	HR(95%CI)	*p*	HR(95%CI)
**Univariate analysis**				
Age (< 60years)	0.232	21.827 (0.139–3436.270)	0.122	1.454 (0.905–2.336)
Gender (male)	0.307	1.311 (0.779–2.207)	0.017	1.780 (1.110–2.853)
Tumor size (5 cm)	0.320	1.282 (0.786–2.089)	0.591	0.886 (0.569–1.378)
Tumor site				
Rectum		Refer		Refer
Left colon	0.484	1.263 (0.657–2.427)	0.672	0.889 (0.515–1.534)
Right colon	0.130	1.631 (0.865–3.076)	0.054	0.590 (0.344–1.010)
Pathological T stage	0.087	1.851 (0.915–3.747)	< 0.001	3.363 (1.620–6.980)
Lymph node metastasis	< 0.001	3.552 (2.141–5.894)	< 0.001	2.447 (1.573–3.807)
TNM stage	< 0.001	4.219 (2.497–7.128)	< 0.001	2.764 (1.259–3.206)
Differentiation	0.054	1.668 (0.991–2.809)	0.003	2.009 (0.991–2.809)
Vessel invasion	0.275	1.321 (0.801–2.180)	< 0.001	2.816 (1.808–4.385)
Intraneural invasion	0.206	1.727 (0.741–4.024)	0.319	1.424 (0.710–2.857)
Tumor deposit	< 0.001	4.138 (2.205–7.769)	< 0.001	3.973 (2.359–6.692)
Colonic perforation	0.500	0.506 (0.070–3.657)	0.763	1.194 (0.377–3.786)
Tumor budding	0.318	1.311 (0.771–2.229)	< 0.001	2.411 (1.453–3.999)
*Schistosomiasis*	0.474	1.225 (0.703–2.132)	—	—
Ulceration	0.212	0.725 (0.437–1.201)	0.744	1.077 (0.691–1.676)
Histological type	0.345	0.685 (0.312–1.503)	0.364	1.343 (0.710–2.538)
CD8 density	< 0.001	0.412 (0.239–0.711)	0.002	0.459 (0.283–0.745)
sPD-L1	0.893	1.035 (0.624–1.717)	0.005	0.494 (0.302–0.807)
tPD-L1	0.823	1.059 (0.639–1.756)	0.197	0.729 (0.452–1.178)
**Multivariate analysis**				
Gender			0.028	1.740 (1.062–2.852)
Pathological T stage	—	—	0.046	2.182 (1.015–4.688)
TNM stage	< 0.001	3.250 (1.836–5.755)	0.036	1.729 (1.035–2.887)
Differentiation	—	—		
*Schistosomiasis*	—	—	—	—
Vessel invasion	—	—	0.080	1.549 (0.950–2.526)
Tumor deposit	0.027	2.106 (1.086–4.084)	0.033	1.935 (1.056–3.545)
CD8 density	0.045	0.592 (0.337–1.039)	0.037	0.574 (0.341–0.966)

— data is non-significant

*Abbreviations*: *CI* confidence interval, *HR* hazard ratio

*p* < 0.05 was defined as the criterion for variable deletion when performing backward stepwise selection

## Discussion

Various tumor entities with elevated immune response have dense CD8 pos T cell infiltrates in common, which are responsible for a local production of interferon gamma (IFNγ) [25, 26]. IFNγ, in turn, provokes the adaptive upregulation of PD-L1 on nearby tumor cells via NFκB [27]. Our results showed that PD-L1 expression in tumoral cells and stromal cells were positively correlated with CD8+ TILs density.

In this study, the expression of PD-L1 in tumor cells and immune stroma were associated with less aggressive tumor features and translated into favorable OS in patients with CRC cancer. These were consistent with J Wyss et al.’s findings [4]. The association of PD-L1 expression with beneficial clinical outcome has been reported in a diverse set of tumor types, such as NSCLC [28], melanoma [29], breast cancer [7, 30], and including CRC [8]. This might seem inconsistent with the immunosuppressive function of PD-L1. However, this might be explained that PD-L1 expression within tumor microenvironment is not only as an immunosuppression factor, but rather acts as a reflection of adaptive antitumor immunity, where tumor-infiltrating lymphocytes are activated in response to tumor antigens. Contrary to our findings, Thompson et al. [13] showed that in patients with locally advanced gastric cancer, tumoral, and stromal PD-L1 expression and CD8+ TILs were associated with unfavorable outcome. These opposite results might explained by the interaction between tumor and tumor-associated stroma and TILs might be different among different tumor types.

Our results showed that CD8 density was also an independent predictor for CRC patients. CD8, which is predominantly expressed on cytotoxic T cells, is a crucial component of the cellular immune system and is pivotal for cell-mediated anti-tumor immune response [31, 32]. Previous studies demonstrated that patients whose tumors contained infiltrating CD8+ TIL showed better survival in non-small cell lung cancer (NSCLC) [33–40]. These results further suggest that PD-L1 expression may reflects an association with a TIL-mediated antitumor inflammatory response, rather than always being associated with tumor immune evasion [7]. Unexpectedly, there were no correlation between CD8+ TILs and PD-L1 and schistosomiasis. It was possible that the patients in the cohort with schistosomiasis are obviously older than patients without schistosomiasis (median age of patients with schistosomiasis was 74 years old and that of patients without schistosomiasis was 64.5 years old, *p* < 0.0001). And the vigor of immunity of older people is weak [41]. In order to confirm this speculation, we excluded patients younger than 60 years old, then to analyze the relationship between schistosomiasis and CD8+ TILs. However, the small percentage of SCRC patients did not allow us to perform further analysis stratified by age. Thus, further works in larger cohort are still needed to investigate the impact of *S. japonicum* on CD8+ TILs density and PD-L1 expression.

Our retrospective study had several limitations. First, we do recognized the limitation of utilizing a TMA approach to assess expression of a biomarker that may only be locally present in samples, raising the possibility of false negatives, which could possibly change the significance of PD-L1 expression in CRC. Second, we speculated that IFNγ which secreted by CD8+ T cells upregulated the expression of PD-L1. However, further studies needed to clarify the association between PD-L1 expression and CD8+ TILs and to determine whether this combination has predictive relevance as a biomarker for selecting individual patients for treatment involving PD-1/PD-L1 blockade or for selection of certain tumor types for development. Third, determination of PD-L1 expression in tumor samples was generally performed by immunohistochemistry using various antibodies. Fourth, the threshold for positivity was not formally assessed.

In conclusion, the results in the present study demonstrated that stomal PD-L1 expression but not tumoral PD-L1 expression in the whole cohort and in the NSCRC set were associated with less aggressive tumor feature and translated into better OS. And the expression of PD-L1 was positively associated with CD8+ TILs density.

## Supplementary Information

**Additional file 1: Sup Fig. 1**. Typical sample of schistosomiasis-associated colorectal cancer, the red arrows indicate schistosome ova (HE, ×100). **Sup Fig. 2**. Determination of cut-off values of CD8 density of TMAs and survival analyses. X-tile analysis of OS was performed using patients’ data collected from the pathological system of the Qingpu District Center for Disease Control and Prevention. The optimal cut-off values highlighted by the black circles in left panels are shown in histograms of the entire cohort (middle panels), and Kaplan-Meier plots are displayed in right panels. . The optimum cutoff value of CD8^+^T cell density were determined by X-tile program, which were 279 (χ2 = 15.538, *p* = 0.0029) cell/mm^2^. CD8^low^ group was defined as CD8+ T cells density < 279, and CD8^high^ group was defined as CD8+ T cells density≥ 279 cell/mm^2^.

## Data Availability

The datasets used and/or analyzed during the current study are available from the corresponding authors on reasonable request.

## Abbreviations

PD-L1: Programmed cell death-ligand 1
TILs: Tumor-infiltrating lymphocytes
CRC: Colorectal cancer
tPD-L1: Expression of PD-L1 within tumor cells
sPD-L1: Expression of PD-L1 in stromal cells
OS: Overall survival
AJCC: American Joint Committee on Cancer
TMA: Tissue microarray
IHC: Immunohistochemical
FFPE: Formalin fixed paraffin-embedded
SCRC: Schistosomal-associated colorectal cancer
NSCRC: Non-schistosomal-associated colorectal cancer
NSCLC: Non-small cell lung cancer
