# Supplementary material for: Correlation between schistosomiasis and CD8+ T cell and stromal PD-L1 as well as the different prognostic role of CD8+ T cell and PD-L1 in schistosomal-associated colorectal cancer and non-schistosomal-associated colorectal cancer
Source: World J Surg Oncol. 2021 Nov 7;19:321. doi: 10.1186/s12957-021-02433-w (PMC8573878; doi:10.1186/s12957-021-02433-w)
Supplement: Supplementary file 1 — Additional file 1: Sup Fig. 1. Typical sample of schistosomiasis-associated colorectal cancer, the red arrows indicate schistosome ova (HE, ×100). Sup Fig. 2. Determination of cut-off values of CD8 density of TMAs and survival analyses. X-tile analysis of OS was performed using patients’ data collected from the pathological system of the Qingpu District Center for Disease Control and Prevention. The optimal cut-off values highlighted by the black circles in left panels are shown in histograms of the entire cohort (middle panels), and Kaplan-Meier plots are displayed in right panels. . The optimum cutoff value of CD8+T cell density were determined by X-tile program, which were 279 (χ2 = 15.538, p = 0.0029) cell/mm2. CD8low group was defined as CD8+ T cells density < 279, and CD8high group was defined as CD8+ T cells density≥ 279 cell/mm2. [file 12957_2021_2433_MOESM1_ESM.docx]

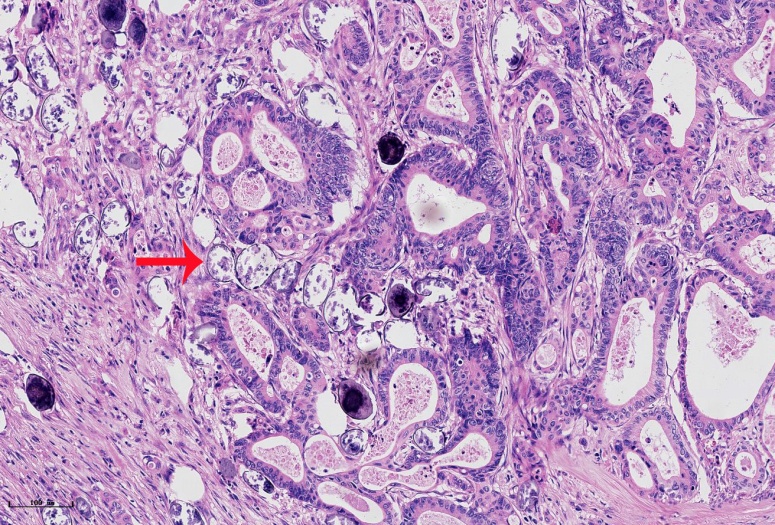


**Sup Fig.1.** Typical sample of schistosomiasis-associated colorectal cancer, the red arrows indicate schistosome ova (HE, ×100)


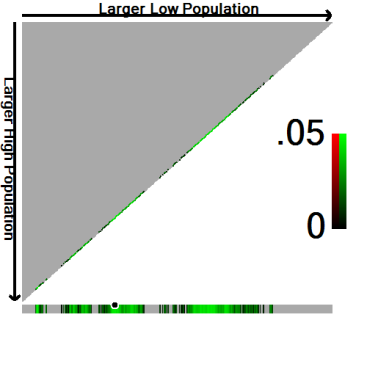

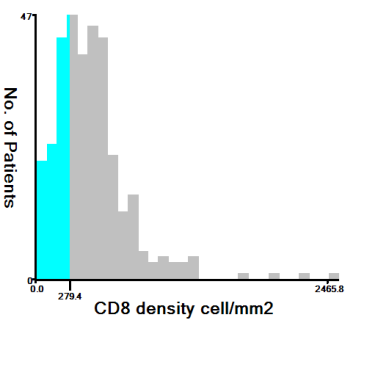

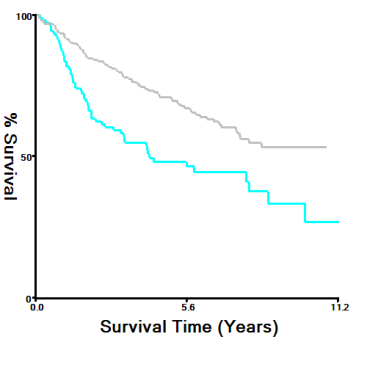


black circle

**Sup Fig. 2.** Determination of cut-off values of CD8 density of TMAs and survival analyses. X-tile analysis of OS was performed using patients’ data collected from the pathological system of the Qingpu District Center for Disease Control and Prevention. The optimal cut-off values highlighted by the black circles in left panels are shown in histograms of the entire cohort (middle panels), and Kaplan-Meier plots are displayed in right panels. . The optimum cutoff value of CD8^+^T cell density were determined by X-tile program, which were 279 (χ2 = 15.538, P = 0.0029) cell/mm^2^. CD8^low^ group was defined as CD8+ T cells density＜ 279, and CD8^high^ group was defined as CD8+ T cells density≥ 279 cell/mm^2^.
